# A deterministic compartmental model for the transition between variants in the spread of Covid-19 in Italy

**DOI:** 10.1371/journal.pone.0293416

**Published:** 2023-11-14

**Authors:** Mario Saviano, Annalisa Fierro, Antonella Liccardo

**Affiliations:** 1 Physics Department, Università degli Studi di Napoli ‘Federico II’, Napoli, Italy; 2 Consiglio Nazionale delle Ricerche (CNR), Institute Superconductors, Oxides and other Innovative Materials and Devices (SPIN), Napoli, Italy; Alexandria Medicine: Alexandria University Faculty of Medicine, EGYPT

## Abstract

We propose a deterministic epidemic model to describe the transition between two variants of the same virus, through the combination of a series of realistic mechanisms such as partial cross immunity, waning immunity for vaccinated individuals and a novel data-based algorithm to describe the average immunological status of the population. The model is validated on the evolution of Covid-19 in Italy, during the period in which the transition between Delta and Omicron variant occurred, with very satisfactory agreement with the experimental data. According to our model, if the vaccine efficacy had been equal against Delta and Omicron variant infections, the transition would have been smoothed and the epidemic would have gone extinct. This circumstance confirms the fundamental role of vaccines in combating the epidemic, and the importance of identifying vaccines capable of intercepting new variants.

## 1 Introduction

About three years after the first wave of the Covid-19 epidemic broke out, our knowledge of the SARS-Cov2 virus has significantly increased, thanks to a vast research activity devoted to the comprehension of the characteristics of the virus from a genomic and immunological point of view (e.g. [1–4]), to the characterisation of the symptomatology induced by the virus (e.g. [5, 6]) and the related therapeutic approaches (e.g. [7]). At the same time an extensive literature, aimed at modelling the epidemic spreading, has been produced, proposing deterministic compartmental models (e.g. [8–11]), focusing on the geographic distribution of the epidemic by means of spatially explicit models (e.g. [12–14]), including appropriate vaccination compartments (e.g. [15–17]).

During this period the virus showed highly variable characteristics, with frequent mutations leading to the appearance of new variants. It also exhibited a waning immunity mechanism, i.e. the rapid decay of immunity, acquired both by contagion and vaccination, that has a crucial impact on the effectiveness of vaccines.

Starting from the wild variant, appeared in Wuhan at the end of 2019, the main variants followed over these years in the world were the Alfa variant (appeared in December 2020, soon becoming dominant), the Delta variant (appeared in India in October 2020, but becoming dominant during the summer 2021) and the Omicron variant (appeared in November 2021 and become dominant at the beginning of 2022), the latter being still dominant worldwide. The Omicron variant, appeared in Italy between late November and early December 2021, completely supplanted the Delta variant in a short time and led to a dramatic regrowth of infections.

Due to evolutionary pressure, a new variant of a pathogen will tend to spread and supplant a previous one if it is able to complete the infection cycle faster and thus can infect a large numbers of individuals compared to others [18]; if it is responsible for a less severe form of the disease (thus inducing infected subjects not to change their habits, for example, by isolating themselves); if it exhibits a fitness advantage because of mutations that permit to escape from both vaccine and natural infection immunity [19]; finally, if *cross-immunity* is in favour of the new variant, that is, if the immunity to the previous variant, acquired by recovering from the new variant, turns out to be more solid than vice versa. If there were no *cross-immunity*, two variants could coexist regardless of their degree of contagiousness.

Modelling the transition between a Covid variant and another is thus not an easy task, because it is governed by a series of factors: not only the previously mentioned cross-immunity, and waning immunity mechanism, but their interplay with the vaccination campaign and other technical and peculiar aspects of the virus.

About vaccinations, is also necessary to keep in mind that, since the beginning of the vaccination campaign in Europe (December 27, 2020 [20]), the EMA (European Medicines Agency) has authorised the administration of five different vaccines (Moderna, Pfizer / BioNTech, Jansen, AstraZeneca, Novavax) with different efficacy and different degree of waning immunity. Furthermore, in Italy, in the time period explored for this work, the vaccination campaign provided for a cycle consisting of three administrations interspersed with a period ranging from one to six months. It is therefore particularly challenging to properly modelize the immune coverage of such a heterogeneous population with respect to the vaccination status, that depends on who, when, with which vaccine and with how many doses has been immunised.

In this context, we propose a compartmental model to analyse the transition between two variants, that includes: a realistic mechanism of cross-immunity, the real trend of the vaccination campaign, the waning immunity mechanism, behavioural changes due to the increased risk awareness of the population in correspondence with increased active cases, and variations in the population mobility in specific time windows, all elements that turn out to be crucial for the correct description of the epidemic evolution. Furthermore, we developed an algorithm that allowed us to incorporate the effect of the vaccinal status evolution of the considered population as a whole, without the introduction of further compartments. The model is validated on the evolution of Covid-19 in Italy, during the period in which it reached its maximum peak of spread, the 2021/2022 autumn-winter season, reproducing the transition between Delta and Omicron variant with very satisfactory agreement with the experimental data.

A retrospective analysis has been performed to clarify the role that the different mechanisms have had in shaping the evolution of the epidemiological curve. We find that the most relevant mechanisms are the awareness mechanism, that induces individuals to increase/relax self-protective measures when the number of active cases increases/decreases, and the reduction of vaccine efficacy against the Omicron variant, without which the epidemic would have gone extinct, whereas the mobility variation have had only a minor effect on the total number of infections.

## 2 Materials and methods

In this section we introduce the compartmental model specifically built to analyse the evolution of Covid-19 in Italy, during the period in which the competition between the Delta and the Omicron variants was observed. In particular, starting from autumn 2021, with the definitive return of students in presence at school and the gradual reduction of smart working, the mobility of individuals has gradually returned to pre-pandemic values. In the late autumn the Omicron variant appeared, exhibiting a greater ability to evade both vaccine-provided immunity and that obtained from previous recoveries to different variants, as well as being inherently more contagious. Thus, in a short time, it completely supplanted the Delta variant, resulting in a decrease in the *R*_*t*_ index of the latter and in a greater contagiousness of the new variant. In the first phase, the Omicron variant was present in Italy mainly in the BA.1(.n) sub-lineages, however, in the period considered, other more contagious sub-lineages, such as BA.2(.n), began to spread causing a new growth in infections.

It should be noted that, before the appearance of the Omicron variant in addition to the Delta, other strains, such as the Beta and the Gamma, circulated in Italy. However, for the present work we will not contemplate their presence, as their impact on the incidence of the Covid-19 can be considered negligible.

### 2.1 The compartmental *SEIJRD* model

Our model includes six main epidemiological states: susceptible (S) (i.e. individuals that can contract the virus), exposed (E) (i.e. individuals that have been infected but are not yet infective), infected (I) (i.e. individuals that can transmit the virus), infected diagnosed (J), (i.e. infected individuals that received a positive swab test), removed (R) (i.e. previously infected individuals that recovered from the disease) and dead (D) (i.e. infected individuals that die). Furthermore, we split some of the compartments in the vaccinated and unvaccinated subgroups, indicated by the index *j*, with *j* = *v*, *u* (respectively vaccinated/unvaccinated), and according to the variant *i* = *d*, *o* (Delta/Omicron, respectively), from which they are possibly infected. The only exceptions concern the dead compartment (because they do not play any further role in the epidemic spreading) and the recovered individuals, that are separated only with respect to the variant, but not to the vaccination status, that is overcome by the immunity conferred by the infection.

In particular individuals are divided in the mutually exclusive classes reported in Table 1 according to their epidemiological status: the vaccinated and unvaccinated susceptible compartments, *S*^*j*^(*t*), (with *j* = *v*, *u*); the exposed compartments, $E_{i}^{j}\left(t\right)$, (i.e. individuals that have been infected but are not yet infective), the infected compartments, $I_{i}^{j}\left(t\right)$, the diagnosed compartments, $J_{i}^{j}\left(t\right)$, referring with *i* = *d*, *o* to Delta and Omicron variants, respectively; the recovered compartment, *R*_*i*_(*t*), from Delta and Omicron variant, with *i* = *d*, *o*, respectively (that includes both unvaccinated and vaccinated individuals); and finally the dead compartment, *D*(*t*) (that includes vaccinated and unvaccinated individuals, previously infected from Delta or Omicron variant). We do not consider demographic birth and (not related to the virus) death process.

**Table 1:** Variables of the model (*j* = *u*, *v*; *i* = *o*, *d*).

*S* ^*j*^	fraction of susceptible individuals
$E_{i}^{j}$	fraction of exposed individuals
$I_{i}^{j}$	fraction of infective not detected individuals
$J_{i}^{j}$	fraction of infective detected individuals
*R*_*i*_	fraction of recovered individuals
*D*	fraction of dead individuals

It should be noted that, in order to avoid a proliferation of compartments, vaccinated individuals are not divided in relation to the number and types of vaccines received: the immune coverage associated with vaccinated individuals is obtained through an appropriate average process that takes into account the vaccination status of the population at each time (see Section 2.2).

**Fig 1:**
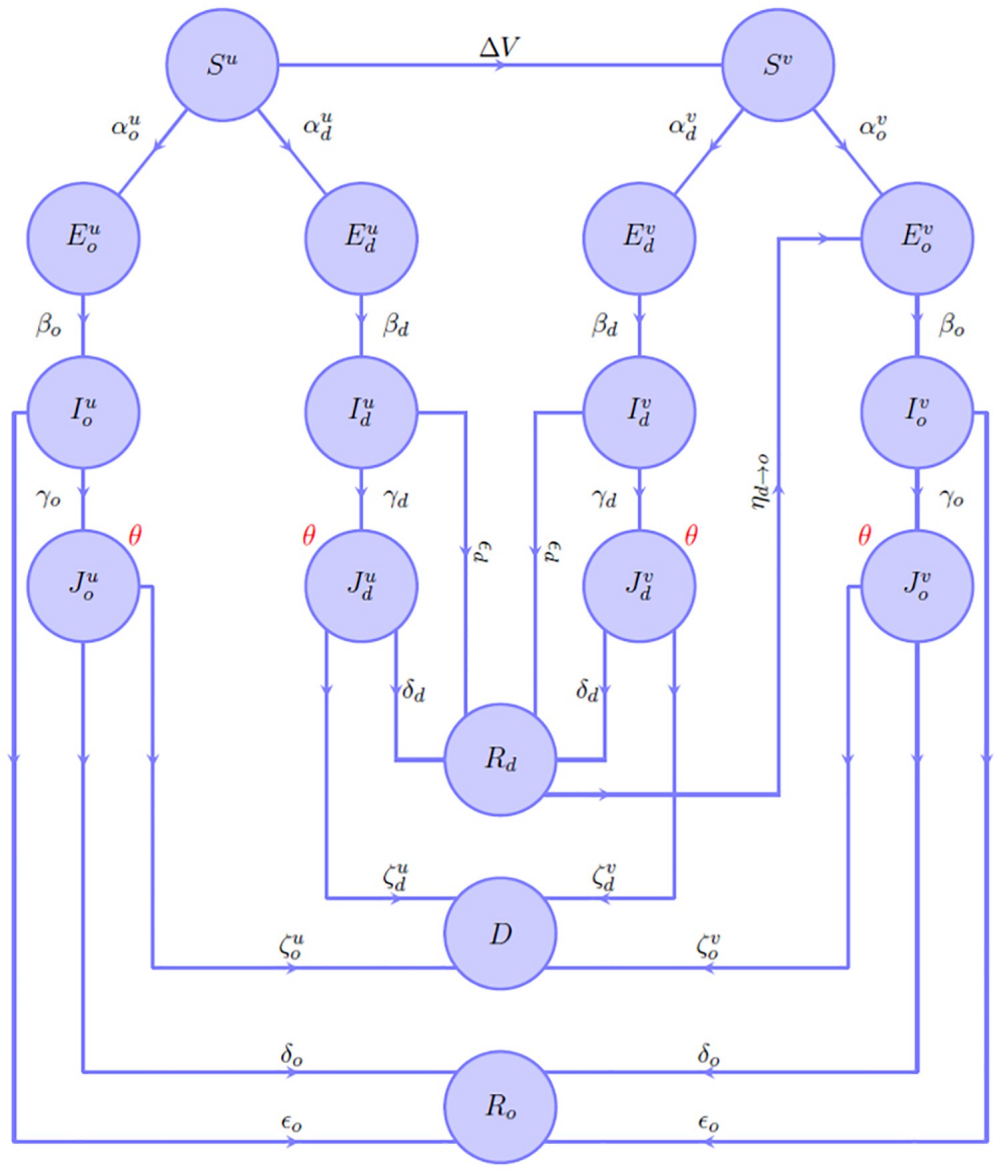
Fluxes diagram.

The dynamics of the model (described by the flowchart in Fig 1) is obtained under the following assumptions (some of which underlying the usual deterministic compartment models):

- The population is closed: new births or deaths not due to the epidemic are not contemplated. Since the time period considered for this work is only five months, it was decided to assume negligible phenomena of immigration/emigration and demographic changes.
- Two variants of the virus, having different characteristics, circulate simultaneously in the population.
- Vaccinated individuals exhibit lower susceptibility to the virus and lower risk of death. Other effects resulting from vaccines (such as slightly shorter recovery times) were deemed less relevant to the spread of the disease and therefore neglected.
- Diagnosed infected individuals are subject to reduced mobility.
- Death can only occur following a diagnosis. This hypothesis is the result of the implicit assumption that the deceased individuals have previously experienced a severe symptomatic picture and that this has certainly led them to the diagnosis of Covid-19.
- The immunity provided by recovering from a specific variant is complete with respect to the same variant and cross-immunity is completely in favour of the new variant. From what is reported in [21], it appears that reinfections attributable to the Delta variant are very infrequent and that those attributable to the Omicron variant, although more likely, occur mainly in the time span ranging from three to twelve months from the previous infection. Therefore, given that the time period considered for this work is only five months and that the Omicron variant began to spread only in this period, it was decided to contemplate as possible reinfections only those due to Omicron variant and only for individuals previously recovered from the Delta variant.
- Only susceptible individuals can receive the vaccine. Again, given the shortness of the time period explored, we assume that the number of vaccine administrations received by cured individuals can be considered negligible when compared to the number of administrations received by susceptible individuals, also taking into account that a recovered individual can receive the administration of a vaccine only after an appropriate time after recovery (at least 120 days [22]).

Under these assumptions, the evolution of the number of individuals (normalised to the total population) present in the various compartments is governed by the following system of ordinary differential equations:

$$\begin{array}{ccc} {\dot{S}}^{u}\left(t\right) & = & -S^{u}\left(t\right)\sum_{i=d,o}\alpha_{i}^{u}\left(t\right)\{I_{i}^{u}\left(t\right)+I_{i}^{v}\left(t\right)+\theta [J_{i}^{u}\left(t\right)+J_{i}^{v}\left(t\right)]\}-\Delta V\left(t\right)\\ \dot{S^{v}}\left(t\right) & = & -S^{v}\left(t\right)\sum_{i=d,o}\alpha_{i}^{v}\left(t\right)\{I_{i}^{u}\left(t\right)+I_{i}^{v}\left(t\right)+\theta [J_{i}^{u}\left(t\right)+J_{i}^{v}\left(t\right)]\}+\Delta V\left(t\right)\\ {\dot{E}}_{d}^{j}\left(t\right) & = & \alpha_{d}^{j}\left(t\right)S^{j}\left(t\right)\{I_{d}^{u}\left(t\right)+I_{d}^{v}\left(t\right)+\theta [J_{d}^{u}\left(t\right)+J_{d}^{v}\left(t\right)]\}-\beta_{d}E_{d}^{j}\left(t\right)\\ \dot{E_{o}^{u}}\left(t\right) & = & \alpha_{o}^{u}\left(t\right)S^{u}\left(t\right)\{I_{o}^{u}\left(t\right)+I_{o}^{v}\left(t\right)+\theta [J_{o}^{u}\left(t\right)+J_{o}^{v}\left(t\right)]\}-\beta_{o}E_{o}^{u}\left(t\right)\\ \dot{E_{o}^{v}}\left(t\right) & = & [\alpha_{o}^{v}\left(t\right)S^{v}\left(t\right)+\eta_{d\to o}\left(t\right)R_{d}\left(t\right)]\{I_{o}^{u}\left(t\right)+I_{o}^{v}\left(t\right)+\theta [J_{o}^{u}\left(t\right)+J_{o}^{v}\left(t\right)]\}-\beta_{o}E_{o}^{v}\left(t\right)\\ \dot{I_{i}^{j}}\left(t\right) & = & \beta_{i}\left(t\right)E_{i}^{j}\left(t\right)-[\epsilon_{i}\left(t\right)+\gamma_{i}\left(t\right)]I_{i}^{j}\left(t\right)\\ \dot{J_{i}^{j}}\left(t\right) & = & \gamma_{i}\left(t\right)I_{i}^{j}\left(t\right)-[\delta_{i}\left(t\right)+\zeta_{i}^{j}\left(t\right)]J_{i}^{j}\left(t\right)\\ \dot{R_{d}}\left(t\right) & = & \epsilon_{d}\left(t\right)\sum_{j=u,v}I_{d}^{j}\left(t\right)+\delta_{d}\left(t\right)\sum_{j=u,v}J_{d}^{j}\left(t\right)-\eta_{d\to o}\left(t\right)R_{d}\left(t\right)\{I_{o}^{u}\left(t\right)+I_{o}^{v}\left(t\right)\\ & & +\theta [J_{o}^{u}\left(t\right)+J_{o}^{v}\left(t\right)]\}\\ \dot{R_{o}}\left(t\right) & = & \epsilon_{o}\left(t\right)\sum_{j=u,v}I_{o}^{j}\left(t\right)+\delta_{o}\left(t\right)\sum_{j=u,v}J_{o}^{j}\left(t\right)\\ \dot{D}\left(t\right) & = & \sum_{i=d,o}\sum_{j=u,v}\zeta_{i}^{j}\left(t\right)J_{i}^{j}\left(t\right), \end{array}$$ (1)

whose variables and parameters are listed in Tables 1 and 2, respectively (superscripts *u*, *v* are used to distinguish unvaccinated from vaccinated individuals, while subscripts *d*, *o* differentiate the Delta from the Omicron variant).

**Table 2:** Model parameters (*j* = *u*, *v*; *i* = *o*, *d*).

$\alpha_{i}^{j}$	infection rates
*β*_*i*_	1/mean latent periods
*γ*_*i*_	detection rates
*ϵ*_*i*_	recovery rates for undetected infected individuals
*δ*_*i*_	recovery rates for detected infected individuals
$\zeta_{i}^{j}$	mortality rates
*η*_*d*→*o*_	re-infection rate
Δ*V*	fraction of individuals passing from the unvaccinated to the vaccinated compartment in the unit time
*θ*	mobility reduction for detected individuals

### 2.2 Model parameters

In this section we will analyse the functional forms attributed to the parameters in Eq (1). Where possible, parameters have been set a priori (see Table 3); conversely, they have been obtained as fit parameters (see Table 4). Time is initialised to the first day of the considered time window (November 15, 2021), therefore, the variable *t*, will identify the time elapsed since that day.

- **Infection rates**, *α*^*u*^(*t*). The infection rate for unvaccinated individuals is factorised as follows
  $$\begin{array}{ccc} \alpha_{d}^{u}\left(t\right) & = & \alpha_{d}^{0}\cdot \alpha^{C}\left(t\right)\cdot \alpha^{A}\left(t\right),\\ \alpha_{o}^{u}\left(t\right) & = & \alpha_{o}^{0}\cdot \alpha^{C}\left(t\right)\cdot \alpha^{A}\left(t\right)\cdot \alpha^{BA.2}\left(t\right), \end{array}$$ (2)
  where
  - $\alpha_{i}^{0}$ are fit parameters (see Table 4);
  - the contact term *α*^*C*^(*t*) and the awareness term *α*^*A*^(*t*) are time dependent functions accounting for the number of contacts among individuals and the effect of preventive measures (imposed by institutions, or self-administrated), respectively.
  - *α*^*BA*.2^(*t*) is a time dependent function accounting for increased infectivity due to the emergence of new sub-lineages BA.2(.n).
  First, it should be noted that the ratio, $\alpha_{o}^{0}/\alpha_{d}^{0}$, represents the probability that a contact between an unvaccinated individual and an infected with Omicron variant (in sub-lineages BA.1(.n)) results in an infection, compared to the same quantity evaluated for the Delta variant. With the values used for the parameters, $\alpha_{d}^{0}$ and $\alpha_{o}^{0}$, shown in Table 4, the ratio, $\alpha_{o}^{0}/\alpha_{d}^{0}$, is consistence within the errors with the value reported in Ref. [28].
  The term, *α*^*C*^(*t*), has been introduced to contemplate the time variation in contacts between individuals due to specific events. In the specific case, the recurrence of the Christmas holidays during the period analysed, certainly modified the type and frequency of close contacts among individuals. It is reasonable to assume that during Christmas holidays individuals are subject to a greater number of contacts, as well as to a diversification of the latter. In addition, it is likely that a climate of greater conviviality increases the likelihood that a contact results in an infection. To mimic such a temporary increase in the rate and proximity of contacts, we shape the term, *α*^*C*^(*t*), as a Gaussian function that takes values greater than one in the interval corresponding to the holidays. More specifically, a good adherence with experimental data was observed by modelling the term *α*^*C*^(*t*) as follows:
  $$\begin{array}{c} \alpha^{C}\left(t\right)=1+K_{c}\cdot e^{-{\left(\frac{t-t_{c}}{\tau_{c}}\right)}^{2}} \end{array}$$ (3)
  and giving the parameters *K*_*c*_, *t*_*c*_, and *τ*_*c*_ the values in Table 4.
  The term, *α*^*A*^(*t*), called *awareness term*, was introduced to implement the effect of precautionary measures taken by individuals in relation to perceived risk. The use of functions to model awareness mechanisms is quite common for these types of models (see for example [29, 30]): as infections increase, it is reasonable to assume that a sense of concern spreads among the population and that health institutions adopt measures to contain the spread, for example through awareness campaigns (a similar mechanism is also introduced in [31]). Moreover, the higher the daily incidence/prevalence, the higher is the probability for susceptible individuals to get in contact with infected ones, and it is desirable that a individual, who knows to have been in contact with an infected one, even if not infected, reduce her/his mobility until the reception of a negative diagnosis.
  It is therefore clear that the term *α*^*A*^(*t*) must be constructed in such a way as to result in a decrease in infection rates in response to an increase in prevalence. In order to exhibit the functional form attributed to the term of awareness we introduce the following function of the generic variable *x* and the parameters *m*, *c*, *w*:
  $$\begin{array}{c} F\left(x;m,c,w\right)=\{tanh[(\frac{c-x}{w})]+1\}\cdot (\frac{1-m}{2})+m. \end{array}$$ (4)
  Given the *w* parameter a positive value, the function *F*(*x*;*m*, *c*, *w*) will be monotonic decreasing in the variable *x* and will assume maximum value 1, minimum value *m* and average value in position *x* = *c*. In addition, the width of the interval between the maximum and minimum value is proportional to *w*. A good adherence with the experimental data was observed by modelling *α*^*A*^(*t*) as a prevalence based function as follows:
  $$\begin{array}{c} \alpha^{A}\left(t\right)=F\left(J^{tot}\left(t-t_{A}\right);m,c,w\right), \end{array}$$ (5)
  (where *J*^*tot*^(*t*) is the observed total prevalence) and assigning to the parameters *t*_*A*_, *m*, *c*, *w* the values shown in Table 4.
  The term, *α*^*BA*.2^(*t*), was introduced to implement an increase in the contagiousness of the Omicron variant due to the spread of BA.2(.n) sub-lineages. Denoted by *χ*(*t*) the relative (observed) incidence of sub-lineages BA.2(.n) and by *ε* the percentage increase in the contagiousness of these sub-lineages compared to BA.1(.n), the function *α*^*BA*.2^(*t*) can be well represented by the following relation
  $$\begin{array}{c} \alpha^{BA.2}\left(t\right)=1+\chi \left(t\right)\cdot \varepsilon , \end{array}$$ (6)
  where *ε* is a fit parameter (see Table 4) and *χ*(*t*) was obtained from the interpolation of the data published by the Italian National Institute of Health (Istituto Superiore di Sanità -ISS) relating to the monitoring of coronavirus variants in Italy (see Table 5).
- **Infection rates**, *α*^*v*^(*t*). Vaccine confers partial immunity to individuals, reducing the probability to get infected during a potentially contagious contact with an infected individual. The infection rates for vaccinated individuals can thus be related to the one for unvaccinated people through the following relations:
  $$\begin{array}{c} \frac{\alpha_{i}^{v}\left(t\right)}{\alpha_{i}^{u}\left(t\right)}=1-\bar{e_{i}}\left(t\right),\;\;\;i=d,o, \end{array}$$ (7)
  where the functions, $\bar{e_{d}}\left(t\right)$ and $\bar{e_{o}}\left(t\right)$, represent the mean effectiveness, at time *t*, of immune coverage provided by vaccines, against Delta variant and Omicron variant infections, respectively. Clearly, the values assumed by these functions at time *t* depend on the vaccination status of the Italian population at the same time. It is thus necessary to give an explicit form to this dependency and then to evaluate the $\bar{e_{d}}\left(t\right)$ and $\bar{e_{o}}\left(t\right)$ evolution in the time period considered for this work. According to [35], the degree of immune coverage of an individual, depends on the types of vaccines received and the administration order. Therefore, in order to evaluate the mean daily effectiveness among individuals of the population, $\bar{e_{i}}\left(t\right)$, one should in principle know when, in what combination, with what frequency and which vaccines each single individual received. Clearly these data are difficult to find and their size would result in a difficult processing.
  However, in Italy the administration of the AstraZeneca and Jansen vaccines were strongly inhibited due to reports of adverse reactions [36–39], whereas the approval of the Novavax vaccine is subsequent [40] resulting, therefore, in a small number of administrations in the period considered. As a result, in Italy, the Pfizer and Moderna vaccines were mainly administered with a ratio of about 4:1 for Pfizer [41]: for this reason and for the fact that the efficacy of the Pfizer and Moderna vaccines are similar, we have decided to approximate the efficacy of all vaccines with the Pfizer one.
  For the calculation of $\bar{e_{d}}\left(t\right)$ and $\bar{e_{o}}\left(t\right)$, we preliminary construct the following functions:
  - *n*_*i*_(*t*, *τ*): number of individuals who, at the day *t*, received the *i*–*th* vaccine dose (and not yet the (*i* + 1) − *th* dose) *τ* days before;
  - *e*_*d*/*o*, *i*_(*τ*): mean efficacy of immune coverage against Delta/Omicron variant infections provided by the *i* − *th* dose of vaccine at *τ* days from the administration (remember that the efficacy of vaccines against coronavirus infections is subject to the “waning” phenomenon, in other words, it tends to decay over time in a more or less pronounced way depending on the peculiar variant of the virus).
  The number of individuals who, up to the day *t*, have received *i* doses of vaccine, which we will indicate below with *N*_*i*_(*t*), can be obtained through the following relationships:
  $$\begin{array}{c} N_{i}\left(t\right)=\sum_{\tau :n_{i}\left(t,\tau \right)\neq 0}n_{i}\left(t,\tau \right). \end{array}$$ (8)
  From the functions just defined it is possible to derive $\bar{e_{d}}\left(t\right)$ and $\bar{e_{o}}\left(t\right)$ through the following relationships:
  $$\begin{array}{c} \bar{e_{d/o}}\left(t\right)=\frac{1}{\sum_{i=1}^{3}N_{i}\left(t\right)}\sum_{i=1}^{3}\sum_{\tau :n_{i}\left(t,\tau \right)\neq 0}n_{i}\left(t,\tau \right)\;e_{d/o,\;i}\left(\tau \right). \end{array}$$ (9)
  In other words, given the number of days elapsed since the start of the vaccination campaign *t*, the mean vaccine effectiveness over the whole Italian population at time *t*, $\bar{e_{d/o}}\left(t\right)$, is evaluated as the weighted mean (with weights *n*_*i*_(*t*, *τ*)) of the efficacies, *e*_*d*/*o*,*i*_(*τ*), against Delta/Omicron variant infections provided by the *i*–th dose of vaccine at *τ* days from the administration, taking into account the waning immunity mechanism. The evaluation of the terms appearing in Eq (9) is demanded to S1 Appendix.
- **Vaccination rate, Δ*V*(*t*)**. Individuals are considered vaccinated if they received at least one vaccine administration. The parameter, Δ*V*(*t*), is thus constructed in such a way to reproduce the pattern of daily administration of first doses of vaccine, obtained by interpolating, the values *n*_1_(*T*, 0) provided by [37] in the time period considered.
- **Mortality rates**, $\zeta_{d}^{u}$, $\zeta_{d}^{o}$, $\zeta_{o}^{u}$, $\zeta_{o}^{v}$
  Following Refs. [25, 26], the efficacy of vaccines against fatal outcomes of the disease is very solid for both variants of the coronavirus, already from the second dose of vaccine. More specifically, individuals, who have received at least two doses of vaccine, are subject to a risk of death due to disease reduced by approximately 80% ÷ 100% (in relation to health status, lifestyle, gender and age) compared to non-vaccinated. Given that in the period considered for this work the fraction of vaccinated population, who received at least two doses of vaccine is always greater than 91%, it was decided to consider the following approximation
  $$\begin{array}{c} \begin{array}{c} \zeta_{d}^{v}=\zeta_{d}^{u}\cdot \left(1-e_{d}^{D}\right)\\ \zeta_{o}^{v}=\zeta_{o}^{u}\cdot \left(1-e_{o}^{D}\right) \end{array} \end{array}$$ (10)
  with $e_{d}^{D}=e_{o}^{D}=90\%$. The rates of mortality of unvaccinated individuals, $\zeta_{d}^{u}$, $\zeta_{o}^{u}$, were instead used as fit parameters and their values, reported in Table 4, denote a mortality of Omicron variant infections reduced by approximately 60% compared to that from the Delta variant, similar to what is reported in [42].
- **Re-infection rate**, *η*_*d*→*o*_(*t*)
  Following what is reported in [27], immunity against the Omicron infections, provided by a recovery to a different variant, is 56.0% (95% CI, 50.6—60.9) about 314 days after the first infection. Since this value is higher than the average efficacy provided by vaccines (calculated in S1 Appendix), it was decided to model the rate, *η*_*d*→*o*_(*t*), as follows:
  $$\begin{array}{c} \eta_{d\to o}\left(t\right)=\alpha_{o}^{u}\left(t\right)\cdot \left(1-e^{R}\right). \end{array}$$ (11)
  with *e*^*R*^ = 56.0% (see Table 3).
- **Recovery rates for detected infected individuals**, *δ*_*d*_ **and** *δ*_*o*_
  The recovery rates for detected infected individuals depend mainly on the time established by the Health Institutions to be allowed to do the healing swab (that was mandatory in Italy, during the period under investigation). Moreover, different regions acted according to different regulations, often subject to revisions. For these reasons, we chose to set *δ*_*d*_(*t*) = *δ*_*o*_(*t*) and to derive them from experimental data.

**Table 3:** Parameters fixed a priori.

*γ*_*d*_ = *γ*_0_	1/3 *days*^−1^	[23]
*β*_*d*_	1/3 *days*^−1^	[24]
*β*_*o*_	1/2 *days*^−1^	[24]
$e_{d}^{D}=e_{o}^{D}$	90.0%	[25, 26]
*e* ^*R*^	56.0%	[27]

**Table 4:** Fit parameters.

$\alpha_{d}^{0}$	0.840 *days*^−1^
$\alpha_{o}^{0}$	0.874 *days*^−1^
*K*_*c*_	1.1
*t*_*c*_	39 *days* (24/12/2021)
*τ*_*c*_	$\sqrt{12}\;\;days$
*t*_*A*_	9 *days*
*m*	0.6
*c*	0.035
*w*	0.02
*ε*	15%
$\zeta_{d}^{u}$	0.00086 *days*^−1^
$\zeta_{o}^{u}$	0.00037 *days*^−1^
*ϵ*_*d*_	1/8 *days*^−1^
*ϵ*_*o*_	1/6 *days*^−1^
*θ*	0.05

**Table 5:** Relative incidence of sub-lineages BA.2(.n) on the total incidence of the Omicron variants.

31/01/2022 (*t* = 77)	3%	[32]
07/03/2022 (*t* = 112)	44.07%	[33]
04/04/2022 (*t* = 140)	86.6%	[34]

## 3 Results

The model in Eq (1) is calibrated on the official data of the Italian outbreak from November 15 to April 4, 2022, reported daily by ISS and publicly available in [43] and has been solved using the Python SciPy libraries, with the functional forms of the parameters illustrated in Sect. 2.2 and the initial conditions reported in S2 Appendix. Input data and code are reported in [44]; simulation data in [45]. The parameters set a priori are listed in Table 3, and the best fit parameters in Table 4. The last one were obtained by minimising the *χ*-square with respect to the incidence experimental data, with the exception of the parameters *ζ*, that make also use of data on dead individuals. We perform a fit procedure on successive time windows. Indeed, different transmission parameters dominate in different time windows and, thus, the best-fit necessary to fix them are performed in progressively subsequent periods. The parameters are fixed, once and for all, in the period in which they are dominant, and maintained constant in the subsequent ones.

**Fig 2:**
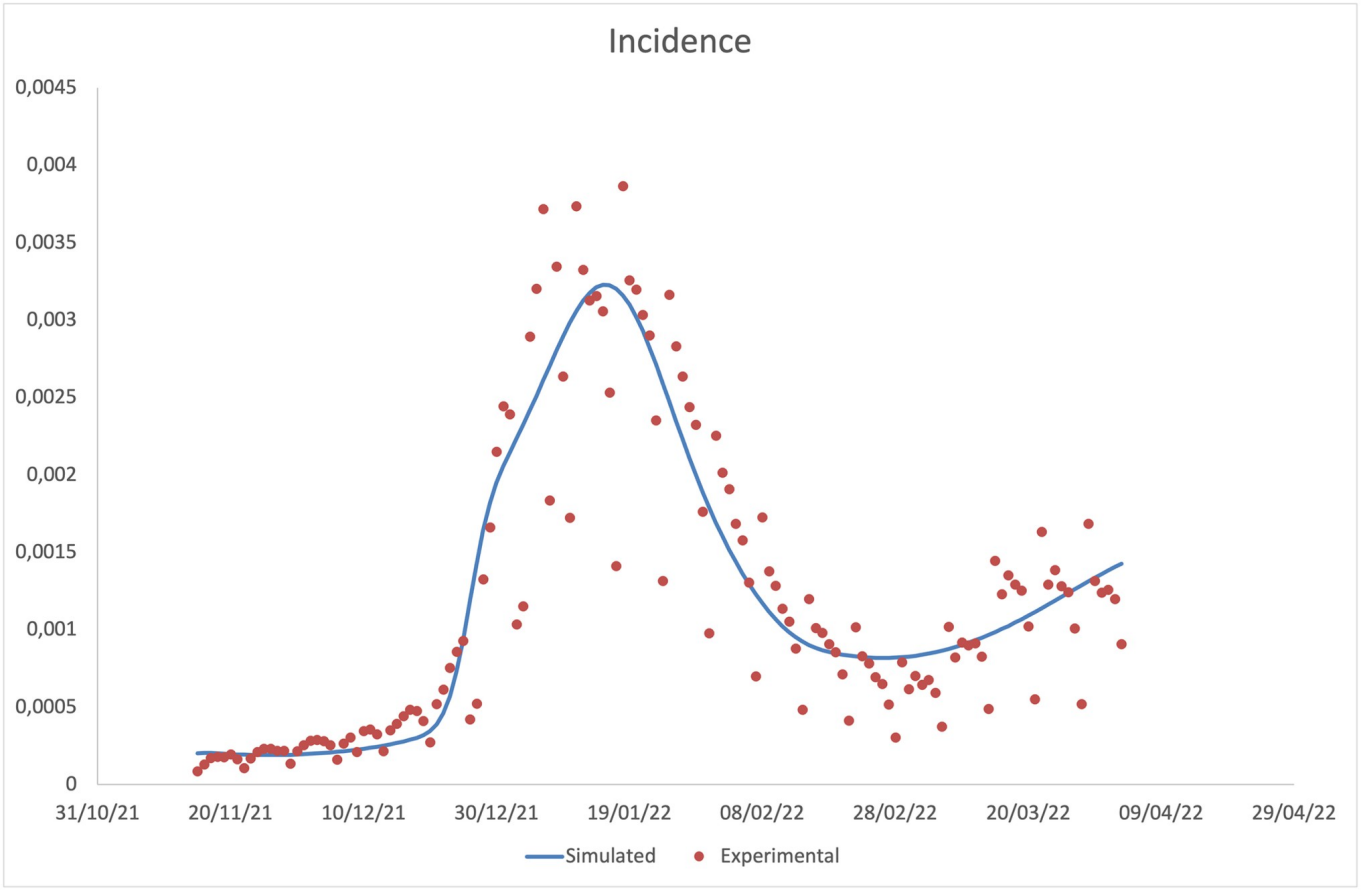
Comparison between the daily incidence predicted by the model and that observed experimentally.

**Fig 3:**
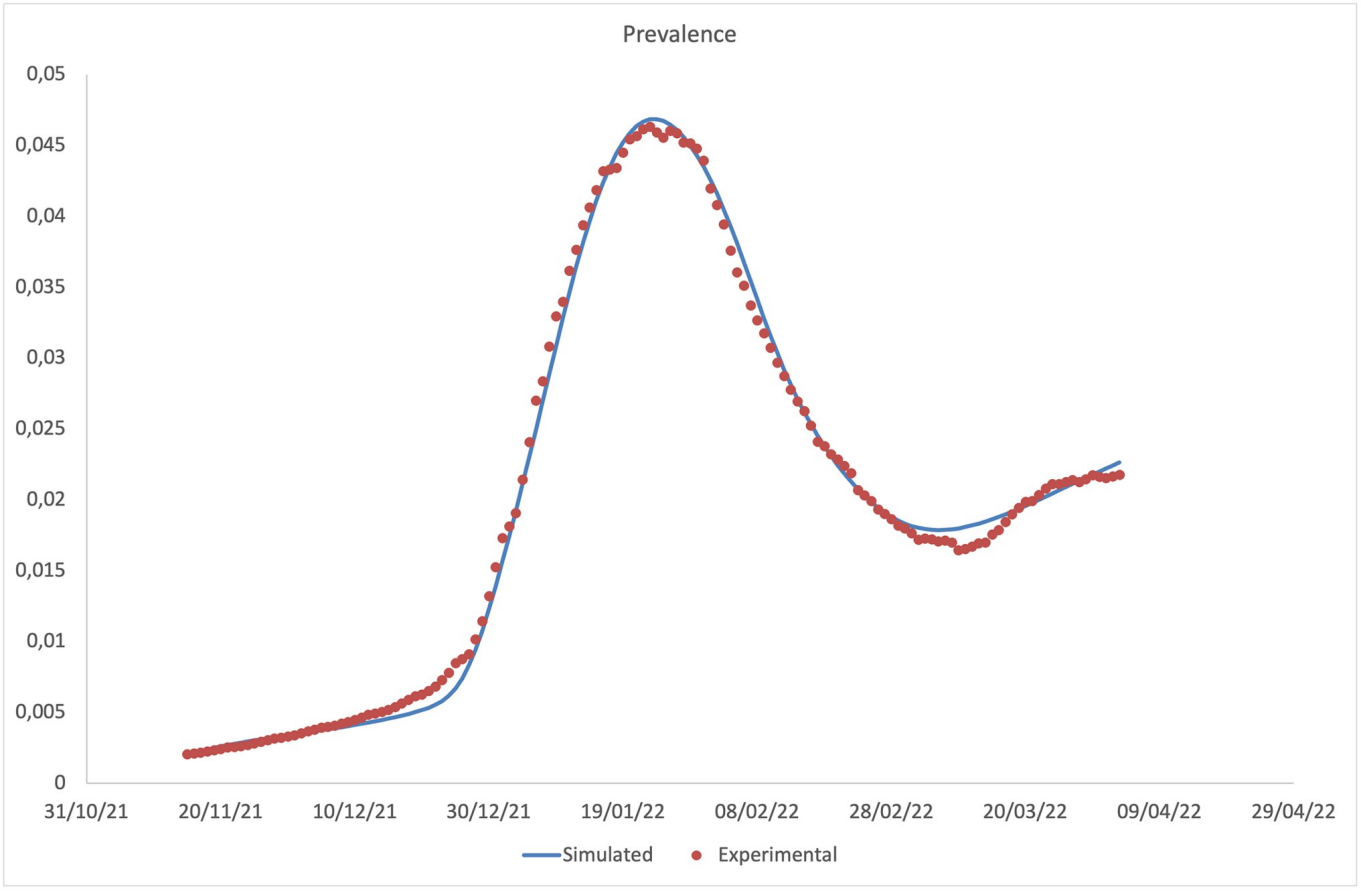
Comparison between the daily prevalence predicted by the model and that observed experimentally.

**Fig 4:**
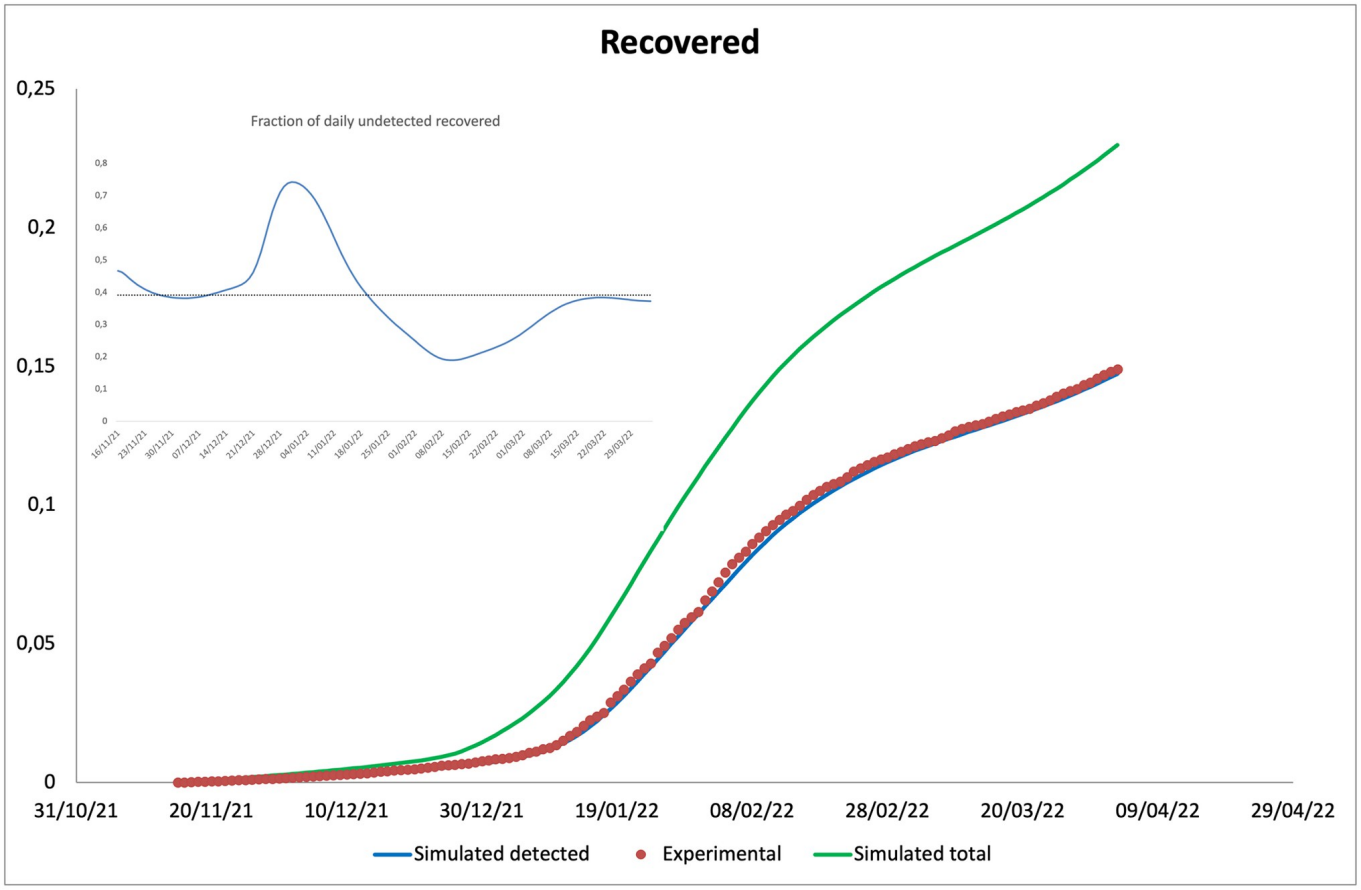
**Main**: Comparison between the cumulative number of recovered individuals (from November 15, 2021) predicted by the model (detected and total) and that observed experimentally. **Inset**: Fraction of undetected recovered individuals. Dashed line represents the mean value.

**Fig 5:**
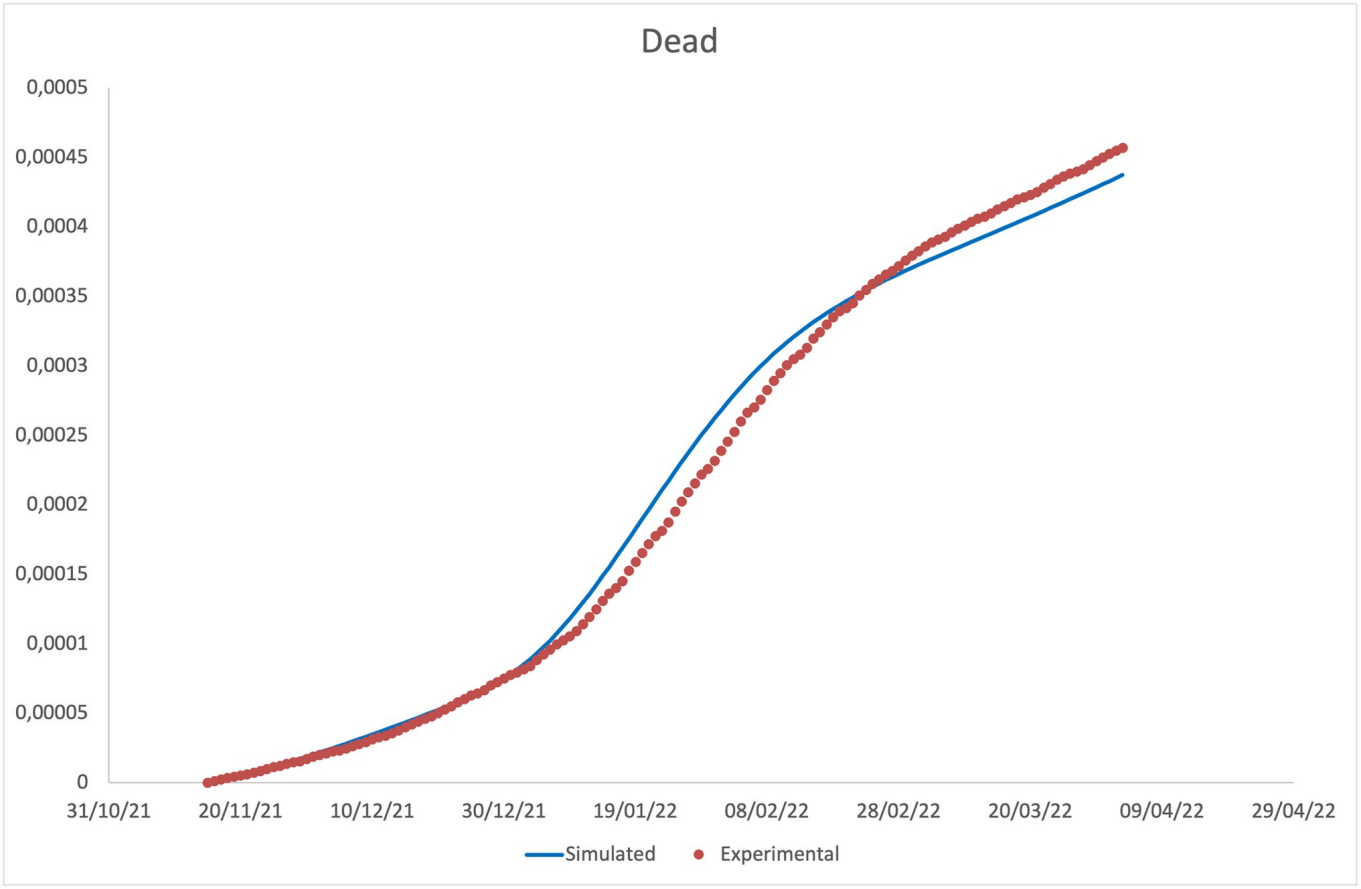
Comparison between the cumulative number of deaths (from November 15, 2021) predicted by the model and that observed experimentally.

In this section, the predictions of our model are compared with the experimental data (normalised with respect to the number of inhabitants of the Italian population, assumed equal to 59.030.133 [46]), provided by the ISS [43]. In Figs 2–5, data for incidence, prevalence, cumulative numbers of recoveries and deaths are respectively plotted. Clearly the experimental data coincide with diagnosed infections, therefore, we evaluated the incidence as the sum of the incoming flows of the compartments $J_{i}^{j}$ (*i* = *d*,, *o*,,, *j* = *u*,, *v*); the prevalence as the sum of the individuals present in the compartments $J_{i}^{j}$ (*i* = *d*,, *o*,,, *j* = *u*,, *v*); the cumulative numbers of deaths and recovered as the integrals over time of flows leaving the compartments $J_{i}^{j}$ (*i* = *d*,, *o*,,, *j* = *u*,, *v*) and entering the compartment *D*, and the compartments *R*_*d*_ and *R*_*o*_, respectively.

It should be noted that the main result is the agreement between the experimental data and the model predictions on the incidence. The agreement on daily prevalence and recoveries is indeed further optimised, by virtue of the choice to derive the recovery rates for diagnosed individuals from the experimental data. Our predictions on cumulative number of deaths, plotted in Fig 5, are instead not in an equally good agreement with the experimental data, showing a slight overestimation at intermediate time and a slight underestimation at long time. We attribute this finding to the choice of fixing a time independent mortality rate for the Omicron variant (being the two disagreement regions both in the Omicron dominated period) within a model without any age-stratification. Indeed, fatality rates are highly dependent on the age of the infected individuals, being higher for elderly ones. Thus, by using a mortality rate not depending on time, we overestimate the deaths when the average age of infected individuals decreases (as it happened during the Omicron peak), and underestimate them when the average age increases (as in the final part of the epidemic curve). Looking at the ISS data on the distribution of the infected individuals among age-classes, we get confirmation of our conjecture. A model without an age stratification, as the present one, and with a time independent fatality rate cannot take in account this aspect.

The data provided in [43], related to the incidence, are not broken down in relation to the particular variant of the coronavirus. However, it is possible to have an indication of the relative incidence of the two variants from the periodic reports [32, 47, 48], provided by the ISS, monitoring the spreading of variants in Italy, where the findings of genomic sequencing (notified respectively on December 20, 2021, January 3 and 31, 2022) are given. From these data an estimation of the relative incidence of the Omicron variant, defined as the ratio between the number of sequences attributable to this variant and the number of total sequences, can be obtained. Compared with those provided by the model (see Table 6), they turn out to be in good agreement only when the relative incidence of the Omicron variant is close to one. This finding is not surprising since, in this limit, both estimates are more reliable. Indeed, in the first weeks, in which the Omicron variant appeared in Italy, the number of sequencing was extremely small and the sequences were not analysed homogeneously throughout the Italian territory, affecting in this way the estimate reliability. For instance, Omicron outbreaks could be undetected in some regions, where there were few or no sequencing. Also the model predictions (which neglects any territorial differences in the circulation of the variants) are less reliable in this limit, where the fluctuations between different regions are not negligible at all.

**Table 6:** Estimates of the relative incidence of the Omicron variant obtained from the ISS reports and the model.

Date	Omicron relative incidence	
	ISS Report	Model
20/12/2021	21.0%	56.6%
03/01/2022	87.5%	89.2%
31/01/2022	99.1%	97.7%

**Fig 6:**
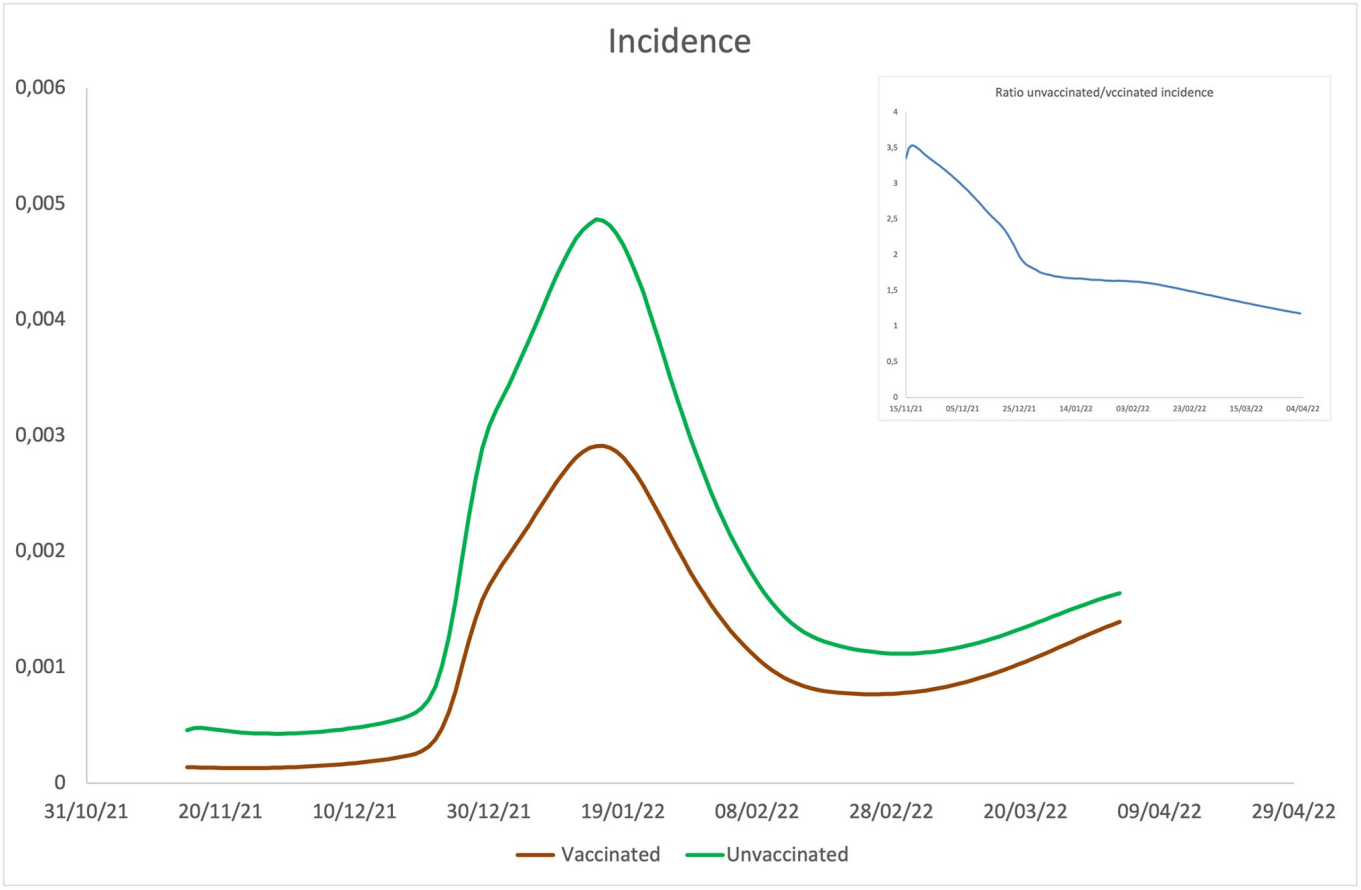
**Main**: Trends (predicted by the model) of incidence in the vaccinated population and the unvaccinated population by number of individuals belonging to the two subgroups. **Inset:** ratio between unvaccinated and vaccinated incidence.

In Fig 6, the predicted trends for the incidence restricted to vaccinated and unvaccinated subjects, normalised with respect to the number of individuals belonging to these subgroups, are reported. This normalisation was carried out by fixing the size of the vaccinated population as the number of individuals, who at any time received at least one administration of vaccine (having as reference the data reported in [37]), and the size of the unvaccinated population as the remaining part. However, it should be specified that, according to the model, unvaccinated individuals from the *R*_*d*_ compartment can also contribute to the incidence of the vaccinated population through the flow $R_{d}\to E_{o}^{v}$ (see Fig 1). However, for the sake of simplicity, it was decided to omit this flow since this affects only marginally the incidence, in the time window considered.

Although it is no longer possible to make a comparison between the incidence of vaccinated and unvaccinated individuals predicted by the model and the corresponding experimental data (as they are no longer publicly available on the ISS website), the ratio between the incidences is consistent with the one calculated in [23], in the common time window between the two simulations (see inset of Fig 3 of the aforementioned article). Moreover, it is observed that the ratio between the unvaccinated/vaccinated incidences between the Delta dominated phase and the one dominated by the Omicron variant has undergone a significant reduction, consistent with the fact that there is a significant difference between the efficacy of the vaccines for the two variants.

Finally, it is interesting to compare the trend of the cumulative number of total recovered individuals with those recovered after a diagnosis: in this way it is possible to follow the invisible circulation of the virus. As shown in the Inset of Fig 4, our model, with the set of parameters chosen, predicts that the fraction of daily undetected recoveries varied in time with a mean value of 40% of infections run without resulting in a diagnosis, a maximum value of about 75%, corresponding to the period of Christmas holidays, and a minimum of about 20%, corresponding to the peak of incidence, due to the appearance of the Omicron variant. It should be stressed that the previous percentages strongly depend on the values of the model parameters, in particular the detection rate *γ*. However being such parameter constant, we expect the trend shown in Fig 4 to be representative of the experimental one.

**Fig 7:**
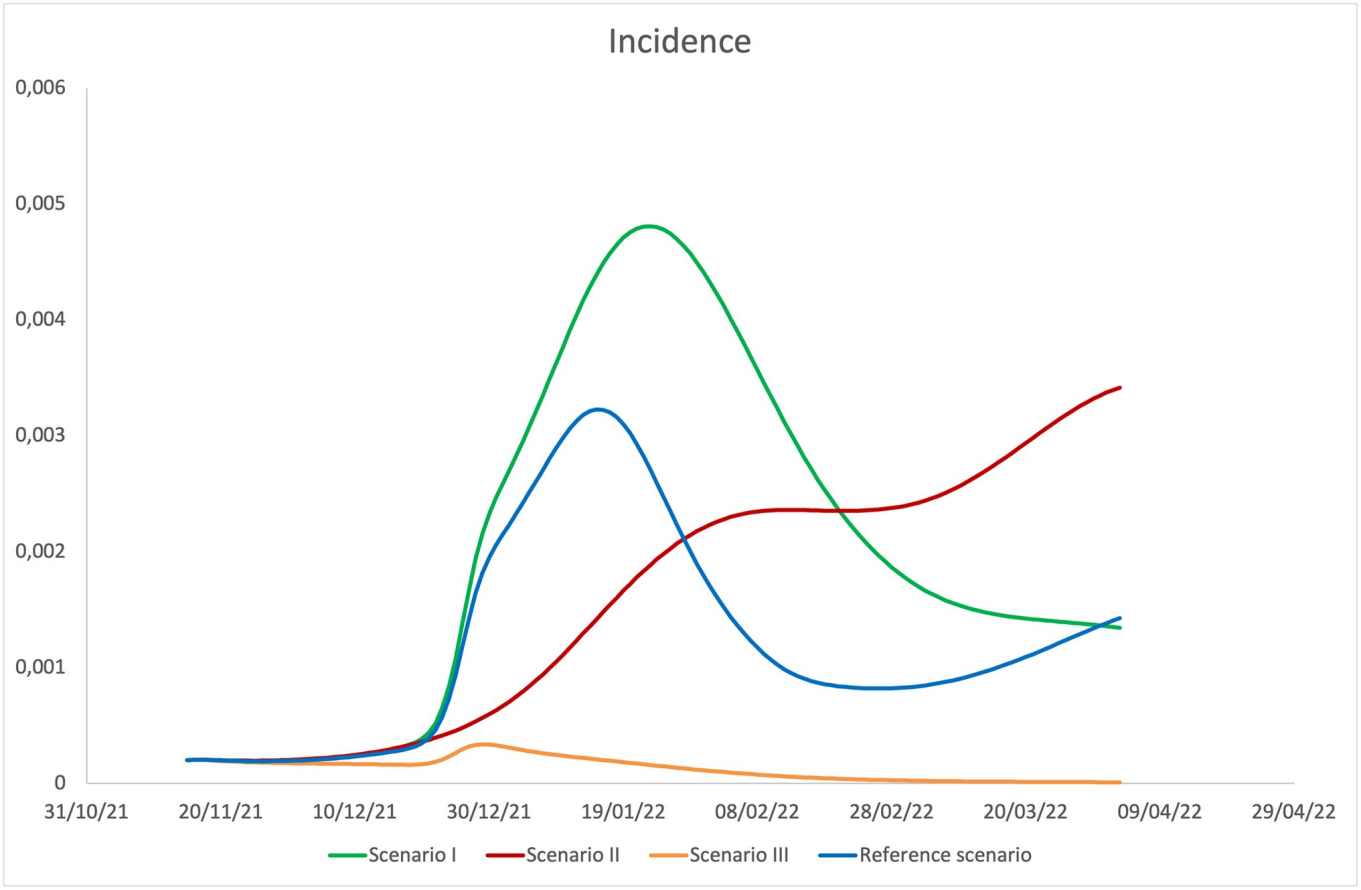
Comparison among the incidence in different scenarios.

To weigh the role that different mechanisms have had in conditioning the evolution of incidence, we considered different scenarios obtained by turning off the mechanism of awareness (scenario I), that of awareness together with that relating to the increase in contacts during the Christmas period (scenario II), and finally that of awareness together with that relating to the reduction of the effectiveness of vaccines against the Omicron variant (scenario III). Fig 7 shows the trend of incidence in each of the scenarios considered. We observe that the mechanism of awareness is crucial in containing the spread of the epidemic according to our model (see Table 7). If this mechanism had not come into play, the cumulative incidence in the time window considered would have increased by 67.5%. It is interesting to note that in scenario II, although there is no Christmas peak, there is a growth trend in the spring phase, realistically attributable to a lower immunity acquired by the population during the previous period. The effect on the cumulative incidence is an increase of 30.7% compared to the baseline simulation. From the analysis of scenario III, it is instead clear that, if the effectiveness of the vaccines had been equally valid against Omicron variant infections, the epidemic would have gone extinct. This circumstance confirms the fundamental role of vaccines in combating the epidemic, and the importance of identifying vaccines capable of intercepting new variants. Summarizing, we find that the most relevant mechanisms are the awareness mechanism, that induces individuals to increase/relax self-protective measures when the number of active cases increases/decreases, and the reduction of vaccine efficacy against the Omicron variant, without which the epidemic would have gone extinct, whereas the mobility variation have had only a minor effect on the total number of infections.

**Table 7:** Percentage variation in the cumulative incidence for different scenarios.

Scenario	Variation% cumulative incidence
I	+67.5%
II	+30.7%
III	-89.5%

It is also interesting to analyse the sensitivity of the model to small parameter perturbations. More specifically, we perturb the parameters reported in Table 3 (i.e. those whose values have been imposed a priori) and the values of the vaccine efficacy, $\bar{e_{d/o}}\left(t\right)$, having as sensitivity indicators the variations in the cumulative numbers of diagnosed infections and deaths (which we indicate respectively with Δ*J* and Δ*D*) over the entire period considered for this work. Tables 8 and 9 show the results of the above analysis.

**Table 8:** Sensitivity of the model with respect to change of 2% in parameters, shown in Table 3.

		Δ*J*	Δ*D*
*γ*_*d*_	+2.0%	−0.05%	−0.7%
	−2.0%	+0.05%	+0.75%
*γ*_*o*_	+2.0%	−6.15%	−4.4%
	−2.0%	+6.3%	+4.5%
*β*_*d*_	+2.0%	0.0%	+0.2%
	−2.0%	0.0%	−0.2%
*β*_*o*_	+2.0%	+2.3%	+1.9%
	−2.0%	−2.3%	−1.9%
$e_{d}^{D}$	+2.0%	0.0%	−0.7%
	−2.0%	0.0%	+0.7%
$e_{o}^{D}$	+2.0%	0.0%	−2.9%
	−2.0%	0.0%	+2.9%
*e* ^*R*^	+2.0%	−0.2%	−0.1%
	−2.0%	+0.2%	+0.1%

**Table 9:** Sensitivity of the system with respect to variation of l 2% of the functions $\bar{e_{d/o}}\left(t\right)$.

		Δ*J*	Δ*D*
$\bar{e_{d}}\left(t\right)$	+2.0%	−0.4%	−2.3%
	−2.0%	+0.5%	+2.7%
$\bar{e_{o}}\left(t\right)$	+2.0%	−7.7%	−4.7%
	−2.0%	+7.9%	+4.8%

**Fig 8:**
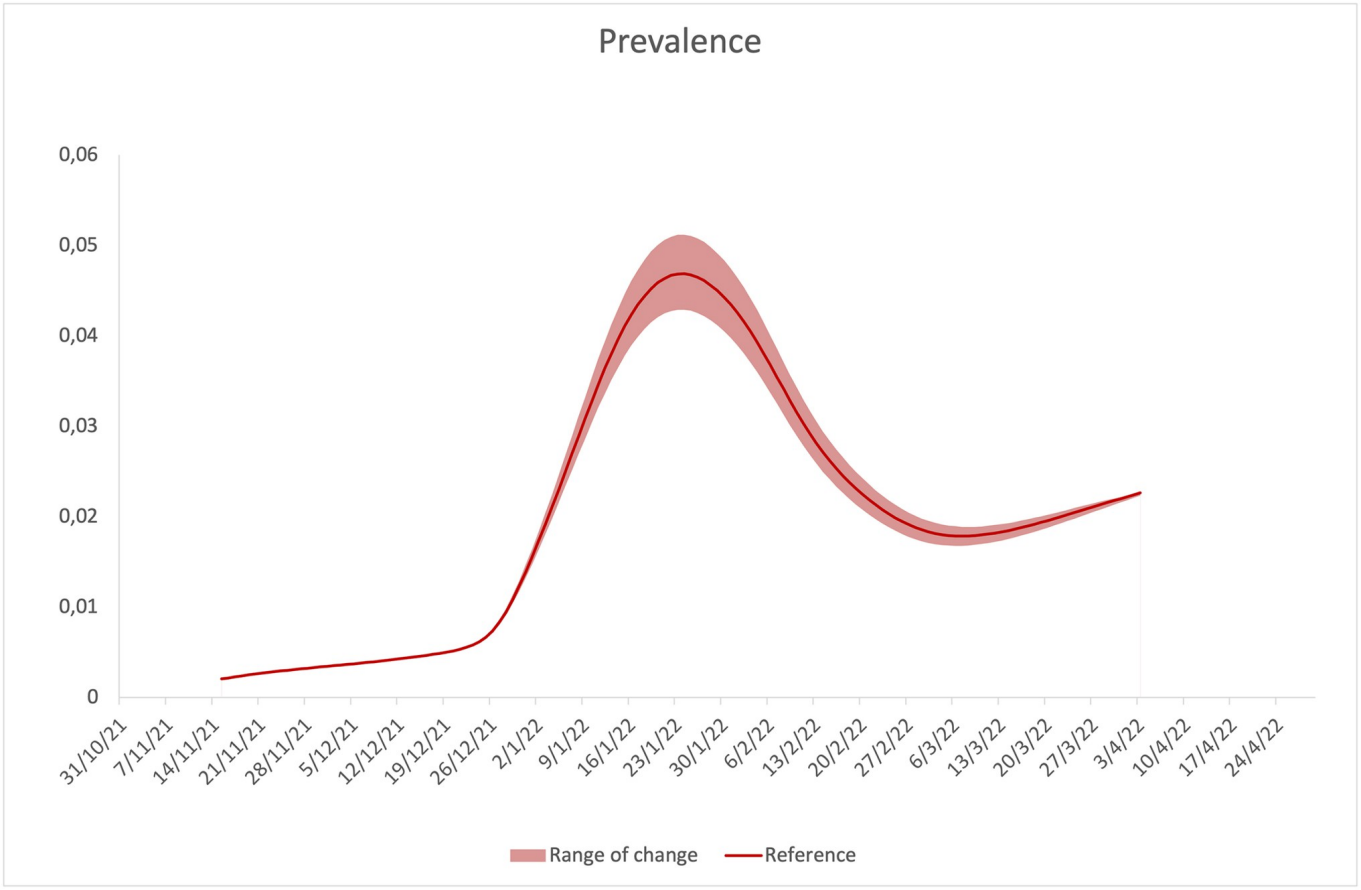
Range of change in prevalence, in response to perturbation of $\bar{e_{o}}\left(t\right)$ in the region $\left[\bar{e_{o}}\left(t\right)\cdot 98\%,\bar{e_{o}}\left(t\right)\cdot 102\%\right]$.

**Fig 9:**
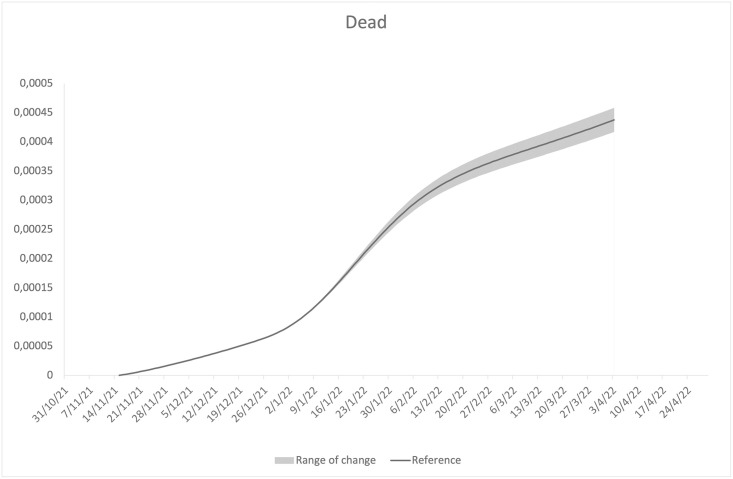
Range of change in cumulative number of deaths, in response to perturbation of $\bar{e_{o}}\left(t\right)$ in the region $\left[\bar{e_{o}}\left(t\right)\cdot 98\%,\bar{e_{o}}\left(t\right)\cdot 102\%\right]$.

In agreement with the fact that the Delta variant is less contagious, but significantly more lethal than the Omicron, perturbations of the parameters related to the Delta variant lead to Δ*D* variations more marked than the Δ*J*. The opposite happens in the case of perturbations of the parameters related to the Omicron variant. Data in tables also show that the model appears to be more sensitive to perturbations of the parameters related to the Omicron variant, and particularly sensitive to perturbations of the detection rate, *γ*_*o*_, and the vaccine efficacy, $\bar{e_{o}}\left(t\right)$. These findings are even more evident in Figs 8 and 9, which show changes in the prevalence and in the cumulative number of deaths, in response to perturbations on the efficacy, $\bar{e_{o}}\left(t\right)$, in the range $\left[\bar{e_{o}}\left(t\right)\cdot 98\%,\bar{e_{o}}\left(t\right)\cdot 102\%\right]$. Changes in response to perturbations on the detection rate, *γ*_*o*_, in the range [*γ*_*o*_ ⋅ 98%, *γ*_*o*_ ⋅ 102%], show trends that are perfectly comparable to the previous ones. From Fig 8, it is also clear that the sensitivity depends on the epidemiological situation, being more accentuated around the peak. In this phase, therefore, the error of any forecasts provided by the model would be particularly accentuated. A similar finding was observed in the Ref. [49].

## 4 Discussion

The insurgence of new variants [50, 51] has been the subject of different multi-strain models, either formulated as network models [52, 53], or as deterministic compartmental models [54, 55].

Table 10 summarises the main characteristics of some models present in literature, compared to the one presented here. In particular, we focus on specific aspects such as the inclusion of some sort of cross-immunity mechanism, the modellization of the vaccination campaign, the inclusion of a waning immunity mechanism for vaccination induced immunity and the break down of the simulated data with respect to different strains, in order to analyse coexistence/transition processes. There are two crucial points in modelling the coexistence/transition between two different variants. The first one is the way in which the cross-immunity is treated (absent, partial, full cross-immunity), the second one is the way in which the immunity decay in time (if is permanent or not). Some papers opt for a full cross-immunity mechanism (i.e. the recovery from one of the two strains confers total immunity with respect to both the strains) [56, 57], however, the explicit transition from one variant to the other is rarely subject of analysis [57]. In particular, the last model focus on the transition occurred in Italy from the wild to the Alpha variant, the second one assumed to be more contagious than the first one, and becoming prevalent in April 2021, with a satisfactory agreement for the 2020 waves of epidemic, and lower accordance in the winter-spring 2020.

**Table 10:** Comparison with other models in literature.

Ref.	Cross immunity	Vaccine	Waning Immunity	Break down
present model	Partial	Yes	Yes	Yes
[56]	Full	No	No	No
[57]	Full	Yes	Yes	Yes
[58]	Absent	No	No	Yes
[59]	Absent	Yes	Yes	No

On the other hand, there are models, such as [58, 59], that do not include any mechanism of cross immunity, in which an individual, previously recovered from any of the two strains, becomes susceptible to both the strains, at constant rate. In particular, in [59] authors propose a compartmental model focused on the dynamics of the Omicron and Delta variants in the United States, which also includes vaccination at a constant rate. The paper reproduces with good agreement the experimental data regarding the cumulative daily cases, without discriminating between Delta and Omicron cases and consequently without actually analysing the transition between one variant and another. On the other hand, the lack of a mechanism of cross immunity makes it plausible a coexistence scenario between the two strains, as shown in Ref. [58].

One of the main novelties of the present work consisted in having explicitly modelized the transition between one variant and another, through a realistic mechanism of partial cross immunity. It is a fact that the occurrence of Omicron infection on individuals recovered from Delta variant is a much more likely event than vice versa. Our model realistically includes partial cross immunity in favour of the Omicron variant, which played an important role in letting it to supplant the Delta variant.

Secondly our model presents an innovative approach aimed at describing, in an efficient and compact way, the temporal evolution of the immune coverage of the entire population, due to the vaccination, which takes into account the timing and evolution of the vaccination campaign and the decay of immunity conferred by vaccines, without the need to introduce any additional compartment in the model.

A possible question that the reader could ask is: which aspects have been crucial for the correct reproduction of experimental data? Certainly the introduction of an awareness term was crucial to the success of the modelling, without this last term it would not have been possible to reproduce the steep descent observed in the prevalence after the peak. The crucial role of awareness in the rapid containment of the epidemiological curve is an element common to many other compartmental models, both stochastic (see for example [31]) and deterministic (see for example [23, 30]).

In the same way, to reproduce the abrupt phase of rise in prevalence, it was essential to introduce a function designed to model a significant increase of infection rates during the Christmas period, clearly due to a greater number of contacts between individuals. Therefore, our model emphasises the important role that the behaviour of individuals has on the evolution of diffusion.

This type of analysis presents some limitations. First of all, it should be stressed that, as in most of the compartmental models with more than 2 or 3 compartments, the set of parameters fixed by the model are not univocally identifiable. Actually, they are not observable parameters: the idea is to exhibit a reasonable set of parameters, that, within the specific model/formulation, allows to reproduce the epidemic evolution.

Some limitations concern the reliability of the experimental data used to validate the model. The symptomatic spectrum of Covid-19 is very wide and this circumstance, together with the great variability in the number of diagnostic tests carried out daily, has certainly meant that the incidence/prevalence observed is an underestimation of the real one: a completely asymptomatic infected individual certainly has a lower probability of being recognised compared with an individual experiencing a more severe symptomatic picture. This circumstance places a limit on the reliability of experimental data, which are used to test the model itself, since they describe only a part of the actual circulation of the virus. On the other hand the absence of information on comorbidities among the individuals who died could lead to an overestimation of death related to the epidemic.

Further limitation of the model, aimed however to avoid the proliferation of compartments, were those of not having considered the distribution by age groups of the population and different levels of severity symptoms. Any extension of the model to include the above aspects does not present conceptual difficulties and may be subject to future developments.

Because of its simplicity, the model can be easily extended to other transitions between different variants, to other countries and even to other infectious diseases with similar modes of contagion. Naturally, such an extension is subordinated to the availability of data concerning the vaccination status of the population and epidemiological data.

In future, it will be interesting to extend the stochastic models proposed in [60, 61] for the pandemic H1N1 to the spreading of Covid-19 during 2021–2022 autumn/winter: in this period indeed the almost total absence of mobility restrictions, which were instead present during the previous waves, would allow to apply the social contact hypothesis [62] in order to reproduce the epidemic spreading.

## 5 Conclusion

The aim of this work was to develop an epidemic model to describe explicitly the transition between two variants of the same virus. In particular we focused on the evolution of the Covid-19 in Italy, during the period in which the transition from the Delta to Omicron variant occurred, with the purpose to carry out a retrospective analysis and to understand and weigh the aspects that have most influenced the epidemic spreading. The model developed has proved to be able to reproduce, with a satisfactory agreement, the experimental data relating to the spread of the disease in the time window considered, identifying the moment in which the Omicron variant fully supplanted the Delta and the timing in which the transition occurred. A slight discrepancy in the early stages of the diffusion of the Omicron variant is most likely attributable to the fact that the baseline experimental data are affected by significant errors due to the smallness of the samples analysed and their geographical unevenness.

The retrospective analysis of the model emphasised the importance of a responsible behaviour of individuals, made evident by the deep increase of simulated cases in the absence of the awareness mechanism, as well as the importance of having vaccines capable of intercepting new variants.

Among the new elements of the present work, there are the introduction of a partial cross immunity mechanism and a specific function for the evolution of the vaccination status of the population, incorporating the mechanism of waning-immunity through a media process that has allowed to reduce the number of compartments necessary for the epidemic modelling.

## Supporting information

S1 Appendix The terms appearing in Eq (9) are evaluated. (PDF)

S2 Appendix The initial conditions, used for solving Eq (1), are reported. (PDF)
