# Supplementary material for: A deterministic compartmental model for the transition between variants in the spread of Covid-19 in Italy
Source: PLoS One. 2023 Nov 14;18(11):e0293416. doi: 10.1371/journal.pone.0293416 (PMC10645303; doi:10.1371/journal.pone.0293416)
Supplement: S1 Appendix — (PDF) [file pone.0293416.s001.pdf]

Supplementary Information

A deterministic compartmental model for the transition between variants in the spread of Covid-19 in Italy

Mario Saviano, Annalisa Fierro, Antonella Liccardo

Appendix S1 - Average vaccination efficacy parameters

In this appendix, we derive the expressions of $e_{d/o,i}(\tau)$ and $n_i(t,\tau)$, appearing in the average vaccine effectiveness, Eq. (9) (Main Text).

In Ref. [1] the trend over time of the vaccine mean effectiveness, in relation to dose number and combination, is estimated for the English population. Focusing on Pfizer vaccine, we evaluate $e_{d/o,i}(\tau)$, by interpolating the effectiveness values found in Ref. [1]. In Fig. 1, the efficacy, $e_{d/o,i}(\tau)$, of vaccine against Delta and Omicron infections are plotted, for the first, second and third dose, respectively. It is interesting to note that the waning phenomenon is particularly pronounced for Omicron variant. The functions, $n_i(t,\tau)$, completely summarise the vaccine status of population and these would be known only if, for any day, t, the number of received doses and the number of days elapsed since the last administration were known for each individual of the population. In [2] the numbers of daily administrations of first, second and third doses and the daily numbers of individuals, who received one, two and three doses of vaccine, are reported since the start of vaccination campaign, namely $n_i(t,0)$ and $N_i(t)$, respectively. However, we are not able to assign exact values to the functions $n_i(t,\tau)$ for $\tau \neq 0$, and then we resorted to some approximations.

In order to describe our approach, it could be useful to simplify the problem. Let us suppose to have a vaccine with only two doses and to know $n_i(t,0)$, for $i = 1,\ 2$ and for each day, t. Under the hypothesis that individuals, who received the second dose, were those, who had received the first one for longer time, we can define a time $\tilde{\tau}(t)$, such that:

$$\sum_{\tau=\tilde{\tau}(t)+1}^{\infty} n_1(t,\tau) + \Delta n_1(t,\tilde{\tau}(t)) = n_2(t+1,0), \tag{1}$$

where $\Delta n_1(t,\tilde{\tau}(t))$ is a part of $n_1(t,\tilde{\tau}(t))$, suitably chosen in order to reproduce the experimental data. In other words, we are supposing that

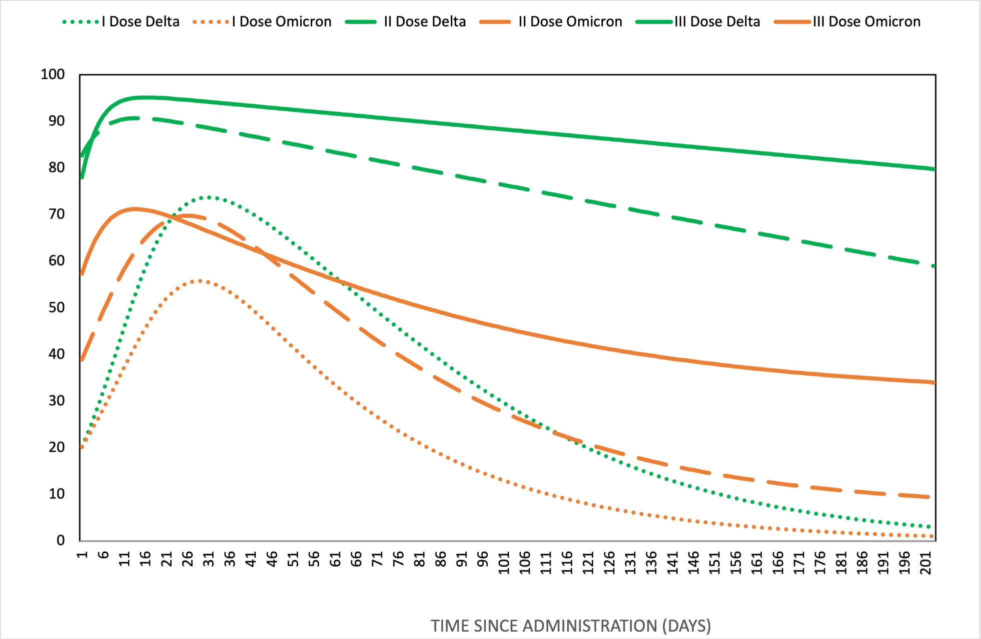

Fig 1. The efficacy, $e_{d/o,i}(\tau)$, of vaccine against Delta and Omicron infections for the first, second and third dose.

individuals, who received the second dose at time $t+1$, are all individuals, who received the first dose at least $\tilde{\tau}(t)+1$ days before, plus a part, suitably chosen, of those, who received the first dose $\tilde{\tau}(t)$ days before. Without any loss of generality, we can define the time interval, $\tau^*(t)$, such that it is not possible to find individuals, who have received the first dose for time longer than $\tau^*(t)$, that are still waiting for the second dose. In this way, Eq. (1) becomes:

$$-\Delta n_1(t,\tilde{\tau}(t)) = \sum_{\tau=\tilde{\tau}(t)+1}^{\tau^*(t)} n_1(t,\tau) - n_2(t+1,0), \tag{2}$$

where $\Delta n_1(t,\tilde{\tau}(t)) < n_1(t,\tilde{\tau}(t))$, and we can write:

$$n_1(t+1,\tau^*(t+1)) = \sum_{\tau=\tilde{\tau}(t)}^{\tau^*(t)} n_1(t,\tau) - n_2(t+1,0), \tag{3}$$

where $n_1(t+1,\tau^*(t+1)) = n_1(t,\tilde{\tau}(t)) - \Delta n_1(t,\tilde{\tau}(t))$. In other words, these are the individuals that at time t have received the first dose $\tilde{\tau}(t)$ days before, but who did not fall among the subgroup who received the second dose at time t. Those will be the first ones to be vaccinated on the next day $t+1$. Thus, according to our simplification, at the next step $t+1$, it will not be possible to find individuals that are waiting for second dose for a time longer than $\tilde{\tau}(t)+1$, i.e. $\tilde{\tau}(t)+1$ will be the new maximum time

occurred since the administration of the first dose at the next step $t+1$, and thus $\tau^*(t+1) = \tilde{\tau}(t) + 1$.

Eq. (3) fully solves our initial problem, since the function $n_2(t, \tau)$ relative to the second dose can be easily obtained from the following recursive equations:

$$n_2(t+1, \tau) = n_2(t, \tau - 1), \quad \forall \tau \tag{4}$$

indeed, the number of new administration of second doses carried out on a given day cannot be changed. Thus those individuals, that at a specific time $t+1$ had received the second dose τ days before, are the same individuals that at time t had received the second dose $\tau - 1$ days before.

For the function $n_1(t, \tau)$, it is necessary to take into account the outflow of those who receive the second dose, so we can write

$$n_1(t+1, \tau) = \begin{cases} n_1(t, \tau - 1), & \tau < \tau^*(t+1) \\ -n_2(t+1, 0) + \sum_{\tau=\tilde{\tau}(t)}^{\tau^*(t)} n_1(t, \tau), & \tau = \tau^*(t+1) \\ 0, & \tau > \tau^*(t+1) \end{cases} \tag{5}$$

Eqs. (4) and (5) are extended to three vaccine doses, which is the cases considered in the present paper, in the following way:

$$n_3(t+1, \tau) = n_3(t, \tau - 1), \quad \forall \tau \tag{6}$$

and

$$n_i(t+1, \tau) = \begin{cases} n_i(t, \tau - 1), & \tau < \tau_i^*(t+1) \\ -n_{i+1}(t+1, 0) + \sum_{\tilde{\tau}_i(t)}^{\tau_i^*(t)} n_i(t, \tau), & \tau = \tau_i^*(t+1) \\ 0, & \tau > \tau_i^*(t+1) \end{cases} \tag{7}$$

where

$$n_i(t, \tau) = 0, \quad \forall\ \tau > \tau_i^*(t), \tag{8}$$

and $i = 1,\ 2$.

References

1. Andrews N, Stowe J, Kirsebom F, Toffa S, Rickeard T, Gallagher E, Gower C, Kall M, Groves N, O'Connell A-M et al. Covid-19 vaccine effectiveness against the Omicron (B. 1.1. 529) variant. N Engl J Med 2022; 386:1532-1546. DOI: 10.1056/NEJMoa2119451

2. Governo Italiano. Report Vaccini Anti COVID-19. https://www.governo.it/it/cscovid19/report-vaccini/

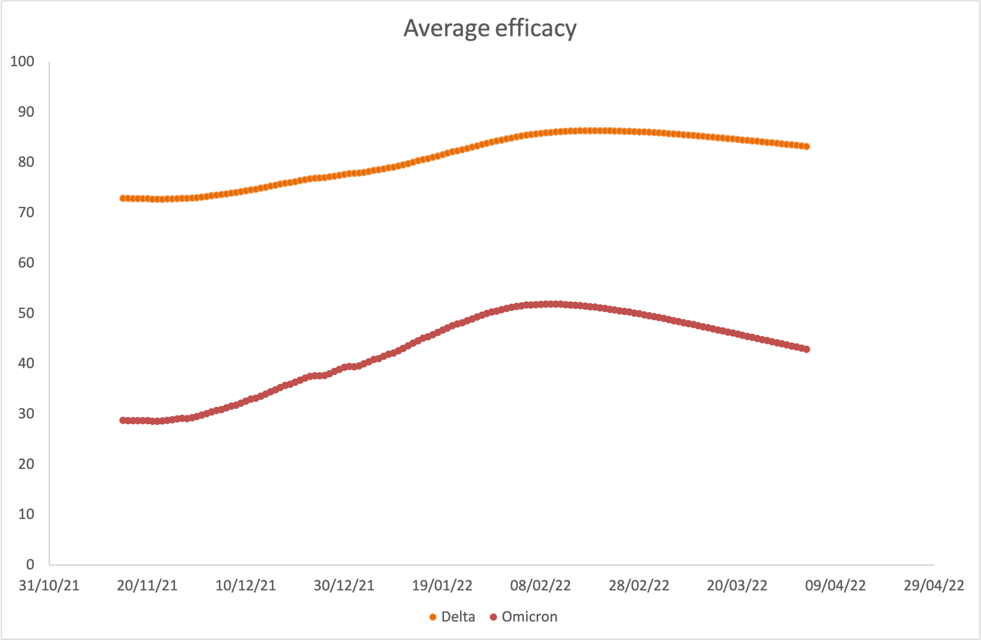

Fig 2. Green: $\overline{e_d}(t)$. Orange: $\overline{e_o}(t)$.
