# Supplementary material for: A deterministic compartmental model for the transition between variants in the spread of Covid-19 in Italy
Source: PLoS One. 2023 Nov 14;18(11):e0293416. doi: 10.1371/journal.pone.0293416 (PMC10645303; doi:10.1371/journal.pone.0293416)
Supplement: S2 Appendix — (PDF) [file pone.0293416.s002.pdf]

Supplementary Information

A deterministic compartmental model for the transition between variants in the spread of Covid-19 in Italy

Mario Saviano, Annalisa Fierro, Antonella Liccardo

Appendix S2 - Initial conditions

Eqs. (1) (Main Text), have been solved using the Python SciPy libraries, with the functional forms of the parameters illustrated in Sect. 1.2 (Main Text) and the initial conditions reported in Table 1. The division of

$S^u(0)$	0.1974771
$S^v(0)$	0.7933164
$E_d^u(0)$	0.0004095
$E_d^v(0)$	0.0004631
$E_o^u(0)$	0.0000400
$E_o^v(0)$	0
$I_d^u(0)$	0.0002600
$I_d^v(0)$	0.0003220
$I_o^u(0)$	0.0000075
$I_o^v(0)$	0
$J_d^u(0)$	0.0010238
$J_d^v(0)$	0.0010238
$J_o^u(0)$	0
$J_o^v(0)$	0
$R_d(0)$	0.0056568
$R_o(0)$	0
$D(0)$	0

Table 1. Initial conditions used for solving Eqs. (1) (Main Text).

infected Delta variant individuals into vaccinated and unvaccinated has been made in such a way as to avoid unexpected fluctuations in the initial stages of pattern-predicted trends in incidence and prevalence relative to the two subgroups of individuals. The initial condition $R_d(0)$ has been fixed as the difference between the cumulative of Covid-19 recoveries observed until 15 November, 2021 and that of recoveries observed until 24 August, 2021, when the Delta variant has become dominant in Italy [1].

References

1. Istituto Superiore di Sanità (2022). Stima della prevalenza delle varianti VOC (Variant Of Concern) e di altre varianti di SARS-CoV-2 in Italia: B.1.1.7, B.1.351, P.1 e B.1.617.2, e altre varianti di SARS-CoV-2 (Indagine del 24/08/2021). https://www.iss.it/documents/20126/0/REPORT+FLASH+SURVEY+Varianti++SARS-CoV-24+agosto+2021.pdf/fcb1c561-15b4-5f33-9d33-d438957b204a?t=1630664243916
